# Breakfast patterns in relation to physical activity among school adolescents in Karachi, Pakistan

**DOI:** 10.1186/s41043-023-00463-6

**Published:** 2023-10-30

**Authors:** Mehak Muhammad Ashfaq, Nida Jawed, Nilofer Fatimi Safdar, Adnan Ahmed Bhutto

**Affiliations:** 1 Department of Human Nutrition and Dietetics, Faculty of Eastern Medicine and Surgery, Hamdard University, Karachi, Pakistan; 2 School of Public Health, Dow University of Health Sciences, OJHA Campus, Karachi, Pakistan; 3 APPNA Institute of Public Health, Jinnah Sindh Medical University, Karachi, Pakistan

**Keywords:** Breakfast, Adolescents, Eating behaviour, Physical activity

## Abstract

**Background:**

Unhealthy habits and poor diet patterns are significant concerns among adolescents, impacting their overall quality of life. This study aimed to assess and improve these habits in adolescents.

**Methods:**

This is a cross-sectional study conducted in 2017–2018 in Karachi. The research participants, aged 11–17 years, were drawn from lower-middle-income secondary schools using multistage random selection. Sociodemographics, the Physical Activity Questionnaire for Adolescents (PAQ-A), and breakfast consumption were determined through questionnaire and a food frequency survey.

**Results:**

A study of 334 school-going adolescents in Karachi, Pakistan, found that 82% consumed breakfast daily, with chapatti being the preferred choice (72.2%). Physical activity levels varied, but 56.6% engaged in regular activity. No significant differences were found in breakfast consumption by age or parental education.

**Conclusions:**

Understanding South Asian adolescents’ breakfast habits is crucial. Promoting healthier breakfast options and increased physical activity are recommended for long-term well-being, with further research needed for targeted interventions.

## Background

Breakfast is considered the most important meal of the day; it plays an integral role in a balanced diet for an individual. Skipping breakfast among adolescents may lead to nutritional deficiencies and poor nutritional status. Information regarding breakfast patterns may help in improving unhealthy breakfast habits and enhance the overall diet patterns of adolescents, thereby improving their quality of life [1].

During the growth and development of an individual from childhood to adulthood, an intermediate period of drastic changes occurs, which is defined as adolescence. It is the most critical period of time that requires intensive mental and physical care for an individual. More specifically, physical growth, mental development, and physiological and psychological changes occur at their peak between the shifts from childhood to adulthood. Ultimately affecting the nutritional demands, while at the same time environmental influences, changes in behaviour, and emotional distress also influence the dietary habits [1].

Nutritional demands are primarily dependent on eating preferences and meals [2]. Anthropological and sociological studies have also examined food patterns and the political economy of health and nutrition in Pakistan. For example, one study found that the food choices of low-income households in Pakistan are often influenced by factors such as poverty, gender inequality, and food insecurity [3]. Various studies have shown that a significant percentage of adolescent’s skip breakfast. According to data from the National Health and Nutrition Examination Survey (NHANES) in the USA, around 20–30% of adolescent’s skip breakfast regularly [4].

Many studies have identified the role of breakfast in enhancing cognition and academic achievements. Regular breakfast is associated with optimal nutritional demand and ultimately satiates the energy demands for mental and physical problem solving, thus improving the level of cognition in those adults [5]. At this phase of life, puberty changes are equally important when considering diet and dietary choices. An increase in body size, muscular development, sexual maturation, and bone metabolism depends on adequate nutrition and physical activity. It is proven that breakfast intake provides energy with enhanced mental activity, focus, and behaviour. Research shows that young children with regular breakfast habits have improved physical activities and learning behaviours [6].

Physical activity is closely linked to breakfast consumption, as it provides the body with the energy and nutrients needed for sustained activity. Skipping breakfast can lead to decreased energy levels, fatigue, and difficulty concentrating, making it more challenging to be physically active [1]. Studies show that adolescents who eat breakfast regularly are more likely to be physically active. A healthy breakfast should include a variety of foods from different food groups, such as fruits, vegetables, whole grains, lean protein, and healthy fats, while avoiding sugary cereals and processed foods [7].

Most of the studies suggest a strong correlation between missing breakfast and increased body weight, increased junk food intake, and a high-fat diet at inappropriate times in the day [8]. Unhealthy behaviours are responsible for skipping breakfast in adolescents, such as smoking, lack of exercise, and a substandard diet [9]. Moreover, unhealthy lifestyles, health-compromising behaviours, and health hazards are also responsible for breakfast skipping among adolescents [10].

Studies reported that adolescents like “ready-to-eat” foods that are already prepared by someone else at home and are usually influenced by choices among peers and other family members. Regarding the food choices of adolescents, other views suggest that cultural trends, socio-economic status, and family influence are major determinants of choice rather than personal perceptions or taste [11].

The discussion focuses on Karachi, the largest city in Pakistan and the sixth-largest in the world by population. Despite its economic and cultural importance, the city faces challenges such as poverty, crime, pollution, and traffic congestion. The Economist Intelligence Unit (EIU) ranked Karachi as the 10th least liveable city globally, affecting stability, healthcare, education, infrastructure, and access to basic services. Access to healthy food is a significant concern for many adolescents in Karachi, compounded by factors like poverty, lack of education, and cultural norms. Nutrition and diet patterns are crucial for health and well-being, and Pakistan's economic situation, health outcomes, and food security influence these patterns [12].

This study investigates breakfast patterns among school adolescents in Karachi, Pakistan, focusing on their relationship with physical activity. It analyses daily consumption, food preferences, and potential links to physical activity, considering sociodemographic factors. The findings can inform targeted interventions to promote healthier habits and physical activity, benefiting the overall well-being of adolescents in the region. Limited research on breakfast consumption patterns among Pakistani adolescents is needed to improve unhealthy habits and enhance overall diet patterns, ultimately improving adolescents' quality of life.

## Methods

### Participants and procedure

In order to account for predicted dropouts and cases with missing data, the sample was inflated by over 30%. Study population was selected using multistage random sampling from six districts of Karachi. One town from each district and a random selection of two schools were made from each of the six randomly chosen union councils (UC). The participants, aged 11–17 years, grades 6–10th, were enrolled based on probability proportional to the enrolment size of each class and school after getting permission from the school administration. Before the sample was finalized, parental written approval was acquired. Strict confidentiality was maintained before the data collection. The study was conducted from November 2017 to November 2018. The sample size of 334 adolescents was determined with a 95% confidence interval, calculated using the OpenEpi calculator.

### Inclusion and exclusion criteria

This study involved students aged 11–17 years, both boys and girls, with signed parent consent. To maintain the integrity of the study’s findings and ensure participant safety, specific selection criteria were applied, participants were expected to be in good general health and not have clinically significant medical abnormalities. Exclusion criteria excluded individuals with a history of serious, chronic, or unstable illnesses or medical disorders deemed unsuitable for study participation. This included conditions that could potentially confound the study's outcomes or pose a risk to the participant’s well-being.

### Instrumentation

A pretested questionnaire that was translated into the local language and used to gather the data asked participants for personal information, sociodemographic data, tobacco and betel nut use, and information about their parent’s education were also noted.

The participants' dietary data, anthropometric measures, and food preference data were collected using the 80-item Food Frequency Questionnaire (FFQ) [1]. To assist the participant in remembering how much they were eating, the team created food models from the paper, plastic, and foil that they brought to each school visit. To estimate food consumption amounts, food models were utilized. ‘Never’ or ‘less than one per month’, ‘number of times per month’, ‘number of times per week’, and ‘number of times per day’ were the replies that were noted [13].

The Physical Activity Questionnaire for Adolescents (PAQ-A) were used which consists of a series of questions related to their participation in physical activities. Prior to each anthropometric measurement, the scale was reset to zero. Belts, bulky outerwear, and shoes were taken off. Height was measured with a portable stadiometer (Seca 213). Participants positioned their arms alongside them, aligned their heels, buttocks, and upper back with the scale, and kept their heads in the Frankfort plane while standing on the stadiometer. A portable bioelectric impedance analysis (BIA) device (Omron BF508) was used to assess weight (to the nearest 0.1 kg) and BF% [13].

According to the World Health Organization (WHO) standards, the body mass index (BMI) was utilized to assess children’s weight status. Those falling below the fifth percentile were categorized as 'underweight', while a BMI greater than or equal to − 1 standard deviation (SD) for the same age and gender was considered 'normal weight'. 'Overweight' encompassed BMI greater than or equal to + 1 SD but less than the 85th percentile, and "obese" included BMI exceeding + 2 SD or surpassing the 95th percentile for children of the same age and gender, providing distinct weight status classifications based on BMI measurements [14].

After this, participants were asked to choose their choices from the list of five food groups that are dairy group, grain group, fat group, fruits/vegetable and fruit juices group, and meat and poultry group. The FFQ will also include a specific question to assess breakfast consumption patterns. Breakfast will be defined as any food or drinks consumed before the first school break [1].

### Data analysis

Data analysis was performed using SPSS version 24. Descriptive statistics were calculated for demographic information about children like age, gender, parent’s educational status, physical activities, substance abuse, and BMI. For quantitative study variables, the mean and standard deviation were calculated, and for qualitative study variables, frequency and percentages were computed. The study outcome 'Breakfast consumption' was presented in a bar diagram with frequency and percentages, and the frequency of food item preference was also presented in frequency and percentages. A Chi-square test was applied to observe the statistical significance between breakfast consumption and other study variables, with the statistical significance set at a *p*-value ≤ 0.05.

### Ethical consideration

The study adhered to ethical guidelines, ensuring the confidentiality and privacy of participants’ data. Ethical review board approval from Dow University of Health Sciences was obtained before the commencement of the study.

## Results

A total of 334 participants were included in the study, out of which 29.6% (99) were younger adolescents, and 70.4% (235) were older adolescents, and the gender distribution was 42.5% (142) males and 57.5% (192) females. The demographic characteristics of the study are presented in Table 1. The educational status of fathers showed that 12% (40) of them had no formal education, 7.5% (25) had completed primary level, 4.5% (15) had done secondary level, 19.5% (65) metric, 15.9% (53) intermediate, and 21.6% (72) were graduates.

**Table 1:** Baseline characteristics of study population (*N* = 334)

Variables	*N*	%
Age (years)		
Younger adolescents	99	29.6
Older adolescents	235	70.4
Gender		
Male	142	42.5
Female	192	57.5
Father’s education		
No formal education	40	12
Primary	25	7.5
Secondary	15	4.5
Matric	65	19.5
Intermediate	53	15.9
Graduate	72	21.6
Don’t know	64	19.2
Mother’s education		
No formal education	58	17.4
Primary	30	9
Secondary	26	7.8
Matric	73	21.9
Intermediate	51	15.3
Graduate	51	15.3
Don’t know	45	13.5
Physical activity		
No	87	26
Yes	189	56.6
Sometimes	58	17.4
Substance abuse		
No	200	59.9
Occasionally (< 3/week)	90	26.9
Often (> 5/week)	13	3.9
Everyday	31	9.3
BMI(Kg/m^2^)		
Underweight	69	20.7
Normal	233	69.8
Overweight	32	9.6

While the educational status of the mothers of the study participants showed 17.4% (58) had no formal education, 9% (30) had completed primary education, 7.8% (26) had done secondary schooling, 21.9% (73) were matriculated, 15.3% (51) had done intermediate, and similar numbers had completed graduate studies.

Using PAQ-A, a straightforward question regarding engaging in regular physical activity, participants were asked to rate their physical exercise behaviour. 56.6% (189) of the participants said they engaged in regular physical activity, 17.4% (58) reported they did sometimes, and 26% (87) stated they did not.

BMI categories for participants were determined using BMI values calculated from weight and height measurements. These categories were based on age- and gender-specific BMI percentiles recommended by health organizations like the WHO. Underweight participants fell below the 5th percentile, normal weight individuals were between the 5th and 85th percentiles, and overweight or obese participants were at or above the 85th percentile. Consequently, 20.7% were underweight, 69.8% had a normal BMI, and 9.6% were overweight or obese.

**Fig. 1:**
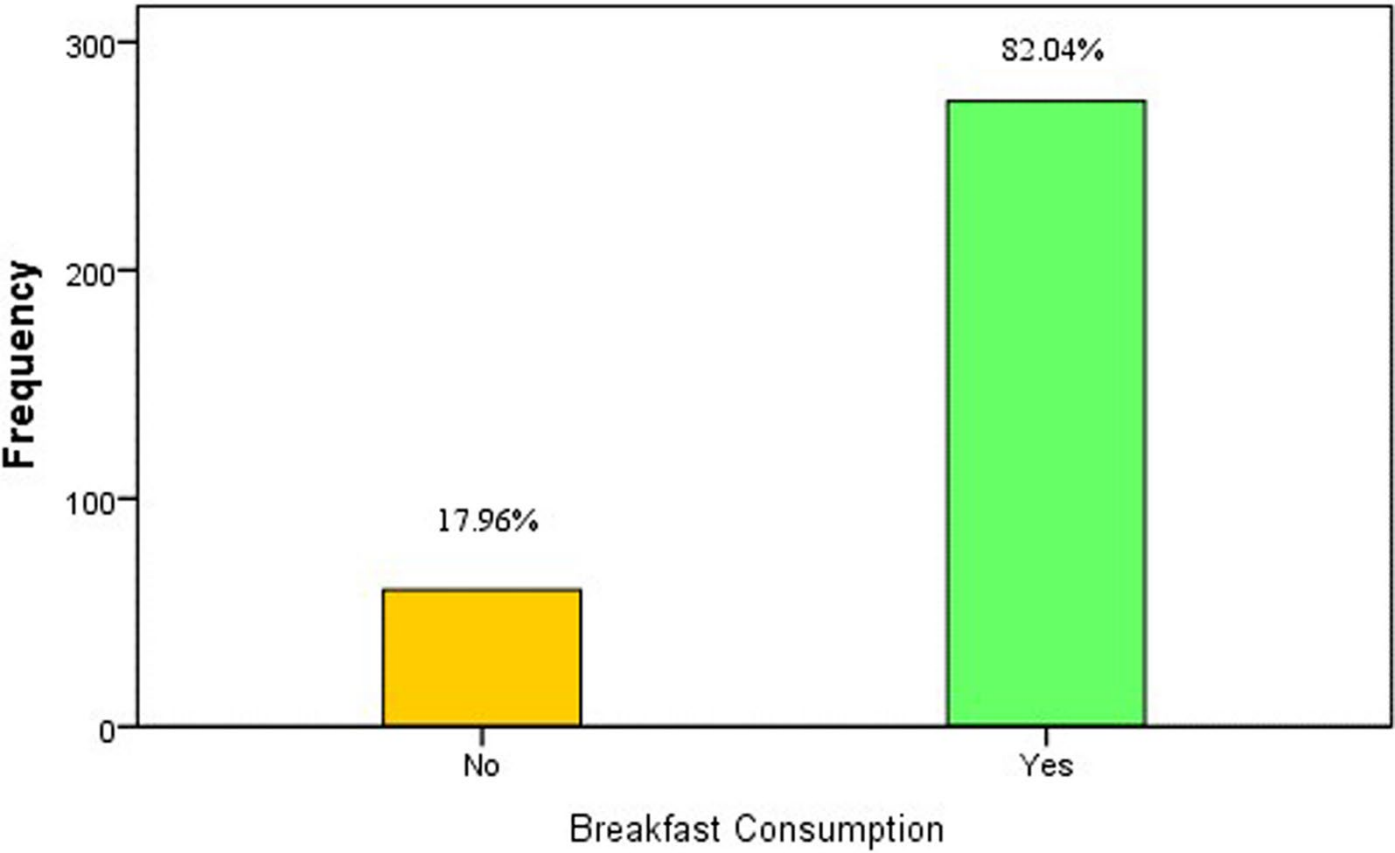
A bar chart with a green bar representing 82% of individuals who consume breakfast, and a yellow bar representing 17.96% who do not

The frequency of daily breakfast consumption was reported by 82% (274) participants, and the remaining did not consume breakfast on a daily basis (Fig. 1). We have assessed the preference of breakfast items according to different food groups among the participants.

The majority of children (72.2%) considered chapatti a primary and main meal for breakfast. 75.7% use baked bread for breakfast. Rusk was a popular choice for breakfast among 60.8% (203) participants. Whole wheat and refined flour were also used by 38.3% (128) and 74.3% (248) children. Puri was consumed by 72.8% (243), porridge by 29.6% (99), cereal by 16.2% (54), beans by 76.3% (255), and lentils by 87.7% (293) were considered a good source of breakfast. In addition to this, tandoori naan was also preferred by 83.2% (278) of participants. The pattern of preference also depended on the socio-economic status of the participant.

In dairy products, whole milk consumption was preferred by 36.2% (121), milk without cream 35.6% (119), milk powder 21% (70), only cream 43.7% (146), yoghurt 80.8% (270), sweet lassi 59.3% (198), and saltish lassi 34.7% (116) of the participant in the study. The most preferred dairy products were yoghurt, milk, and lassi in this study. While in fat, margarine or butter was the only choice, which was preferred by 68.6% (229) participants.

Assessment of preference for consuming fruits and vegetables among the students showed that 69.2% (231 participants) preferred consuming mixed vegetables, 80.5% (269) preferred potato bhujiya, and more than 80% of the students preferred to have fruits in breakfast, the same as for fruit drinks like juices/shakes, etc.

In meat/poultry and seafood, participants who preferred to have red meat in the breakfast, 88% (294), chicken meat was 92.2% (308), fish/seafood was 76.3% (255), and 83.2% (278) of them preferred to keep eggs in their breakfast routine. In other foods, 83.5% (279) participants took bakery items, 86.5% (289) participants consumed tea (brewed), and 60.5% (202) participants preferred using honey/jam or jelly in the breakfast (Table 2).

**Table 2:** Food Item preferences at breakfast (*N* = 334)

Variables	*N*	%
Food group 1: grains		
Chapatti	241	72.2
Bread	253	75.7
Rusk	203	60.8
Paratha whole wheat	128	38.3
Paratha refined flour	248	74.3
Puri	243	72.8
Tandoori naan	278	83.2
Porridge	99	29.6
Cereal	54	16.2
Beans	255	76.3
Lentils	293	87.7
Food group 2: dairy		
Milk whole	121	36.2
Milk without cream	119	35.6
Milk powder	70	21
Cream	146	43.7
Yoghurt	270	80.8
Sweet lassi	198	59.3
Saltish lassi	116	34.7
Food group 3: fat		
Margarine/Butter	229	68.6
Food group 4: fruits/vegetables and fruit juices		
Mixed vegetable	231	69.2
Potato bhujiya	269	80.5
Apple	305	91.3
Banana	302	90.4
Guava	217	65
Grapes	279	83.5
Melon	248	74.3
Orange	282	84.4
Mango	314	94
Pomegranate	268	80.2
Peach	180	53.9
Fresh juice	244	73.1
Fruit drinks	292	87.4
Milk shake	274	82
Food group 5: meat/poultry and seafood		
Red meat	294	88
White meat	308	92.2
Organ meat	187	56
Fish	255	76.3
Egg whole	278	83.2
Miscellaneous		
Bakery items	279	83.5
Tea brewed	289	86.5
Honey/jam or jelly	202	60.5

The frequency of daily breakfast consumption in different age groups varies. 78.8% of younger adolescents and 83.4% of the older adolescent population consumed breakfast daily. There was no significant difference found between both age groups (*p*-value 0.316). Out of the total, 13.4% male participants and 21.4% female participants skipped breakfast, which also showed the non-significant effect of gender on skipping breakfast (*p*-value 0.06). A comparison between breakfast consumption and a parent’s education status showed no significant association (*p*-values 0.285 and 0.793), respectively. 15.9% of participants who were physically active skipped breakfast, whereas 27.6% of those who were sometimes physically active skipped breakfast and 16.1% of those who were physically inactive skipped breakfast in their routine life. Substance abuse also showed an insignificant association (*p*-value 0.592). Body mass index and breakfast consumption status showed that 11.6% of underweight participants skipped breakfast, 79.8% with normal BMI, and 84.4% of overweight participants consumed breakfast daily with an insignificant effect (*p*-value: 0.248). Further, it was observed that skipping breakfast was nearly always associated with being younger, a girl, having low educational status, and having an occasional physical activity pattern (Table 3).

**Table 3:** Frequency of breakfast consumption among all subgroups

Variables	No	Yes	*p*-value
	*N* (%)	*N* (%)	
Age (years)			0.316
Younger adolescents	21.2	78.8	
Older adolescents	16.6	83.4	
Gender			0.061
Male	13.4	86.6	
Female	21.4	78.6	
Father’s education			0.285
No formal education	27.5	72.5	
Primary	16	84	
Secondary	13.3	86.7	
Matric	21.5	78.5	
Intermediate	9.4	90.6	
Graduate	13.9	86.1	
Don’t know	21.9	78.1	
Mother’s education			0.793
No formal education	24.1	75.9	
Primary	20	80	
Secondary	19.2	80.8	
Matric	17.8	82.2	
Intermediate	11.8	88.2	
Graduate	15.7	84.3	
Don’t know	17.8	82.2	
Physical activity			0.11
No	16.1	83.9	
Yes	15.9	84.1	
Sometimes	27.6	72.4	
Substance abuse			0.592
No	17.5	82.5	
Occasionally (< 3/week)	15.6	84.4	
Often (> 5/week)	23.1	76.9	
Everyday	25.8	74.2	
BMI(Kg/m^2^)			0.248
Underweight	11.6	88.4	
Normal	20.2	79.8	
Overweight	15.6	84.4	

## Discussion

A literature review showed that breakfast consumption had a great impact on energy levels, regardless of the food eaten at breakfast. In this study, 82% of our participants consumed breakfast daily. The participants’ preferred breakfast items varied, with chapatti, baked bread, and rusk being popular choices. Dairy products such as yoghourt, milk, and lassi were highly preferred, while red meat, chicken, fish, and eggs were common choices in the meat/poultry and seafood category. The study found no significant difference in breakfast consumption between younger and older adolescents. There was also no significant association between breakfast consumption and parents’ educational status. However, physical activity and body mass index (BMI) showed some non-significant trends, with physically active and overweight participants more likely to consume breakfast regularly.

Our study also sought to explore the relationship between breakfast consumption and physical activity among adolescents. We observed that the majority of participants (56.6%) reported engaging in regular physical activity, while 17.4% were occasional participants, and 26% had no physical activity routine. While the study did not find a statistically significant association between physical activity and breakfast consumption, trends suggest that physically active individuals were more likely to consume breakfast regularly. This observation underscores the potential benefits of promoting both physical activity and healthy breakfast habits among adolescents.

In line with the results of the study, numerous studies around the world also showed breakfast consumption has increased, which might be due to a variety of factors like the availability of different foods for breakfast and awareness from social media, which may have played an important role in having breakfast [15]. On the contrary, a study conducted in the USA showed that breakfast consumption has been reduced over a period of time [16].

Moreover, a survey conducted in Canada also reported that school children skipped breakfast due to a lack of parental education [17]. Similar Japanese studies also reported that the majority of school children did not prefer to take breakfast because they consumed snacks during lunch break [18]. The present study results reveal that the majority of younger adolescents and older adolescent age group prefer to have breakfast daily. However, there is a slight difference between the frequencies of both groups. Another study concluded that 88 per cent of US adolescent tend to skip breakfast more than Australian adolescent [19].

In the current study, more boys consumed breakfast as compared to girls. The results are analogous to a study conducted in Punjab, where more boys tend to eat breakfast as compared to girls [20]. Boys feel hungrier in the morning due to physical activity, while girls are more homebound, leading to less breakfast consumption. This aligns with a study in the Netherlands, which found that females, vocational school students, and minorities consume breakfast less frequently [21].

The study reveals that school children in Pakistan prefer lentils with tandoori naan for breakfast, as they differ from western culture's preference for cereals and whole bread. Additionally, 86% of school children had tea with milk, indicating that tea is more common in the east, while western children prefer semi-skimmed milk. This highlights the cultural differences in food choices among children [21].

Furthermore, the results also showed that sociodemographic factors did not have any effect on breakfast consumption. It can be said that breakfast consumption by school children is entirely an individual’s choice. Hence, the present study did not show any significance [20].

The study on breakfast consumption and physical activity among Pakistani adolescents has limitations, as it did not explore the broader aspects of quality of life. Future research should focus on assessing breakfast habits and physical activity patterns. The study highlights the need for more research on Pakistani adolescents, as breakfast is the most important meal of the day and contributes to a healthy lifestyle. The findings have significant implications for public health policy and interventions, emphasizing increasing awareness of breakfast's importance, providing access to healthy options, and addressing underlying factors like poverty, gender inequality, and food insecurity.

### Implications for school health

Although there are various studies in Pakistan regarding breakfast patterns, the current study showed basic food choices among school children, and there is no significant association with any of the demographic characteristics. School children should be counselled to adopt healthy food choices for breakfast. There should be measures to prevent unhealthy eating during breakfast, and parents should be targeted for this health promotion [22–24].

More focus should be given to breakfast intake among older students in secondary schools, though, as rates of breakfast consumption decline throughout childhood and adolescence. The number of kids skipping breakfast can be greatly reduced through school meal programmes. Families should also be the focus of the campaign because family mealtimes are crucial for developing good eating habits in adolescents. Interventions should emphasize the inclusion of fruit, vegetables, and drinks while encouraging a high-quality breakfast.

### Limitations

The study on breakfast consumption patterns among adolescents has limitations, including a small sample size of 334 participants, self-reported data collection, potential recall bias, and cross-sectional design. It also lacks information on overall dietary intake beyond breakfast, potential confounding factors, and self-selection bias in participation. Despite these limitations, the study provides valuable insights into breakfast consumption among adolescents, but further research with larger, more diverse samples and longitudinal designs is needed to gain a more robust understanding of this important health behaviour. Therefore, we recommend conducting more studies on this crucial topic and promoting health for the benefit of our young population.

## Conclusions

The study provides valuable insights into the breakfast habits of adolescents in this region. The findings indicate that a significant percentage of participants consumed breakfast daily, with chapatti, baked bread, and yoghourt being popular choices. The study did not find significant differences in breakfast consumption based on age or gender, but physical activity and BMI showed some non-significant trends, suggesting a potential relationship between regular breakfast consumption and healthier lifestyle habits. Overall, this study focused on food choices that were not previously studied in Pakistan. However, it is important to acknowledge the study's limitations, such as a small sample size, self-reported data, and cross-sectional design, which may impact the generalizability and causality of the results. Nonetheless, this research serves as a foundational step in understanding the relationship between breakfast patterns and physical activity among school adolescents in Karachi, Pakistan, and underscores the need for further exploration in larger, more diverse samples using longitudinal designs to establish robust conclusions and inform effective interventions promoting healthy behaviours in this population.

## Data Availability

The data that support the findings of this study are available from the corresponding author upon reasonable request.
